# Understanding nutrition, depression and mental illnesses

**DOI:** 10.4103/0019-5545.42391

**Published:** 2008

**Authors:** T. S. Sathyanarayana Rao, M. R. Asha, B. N. Ramesh, K. S. Jagannatha Rao

**Affiliations:** Department of Psychiatry, JSS Medical College, Mysore - 570 004, India; 1 Department of Sensory Science, Central Food Technological Research Institute, Mysore - 570020, India; 2 Department of Biochemistry and Nutrition, Central Food Technological Research Institute, Mysore - 570020, India

## INTRODUCTION

Few people are aware of the connection between nutrition and depression while they easily understand the connection between nutritional deficiencies and physical illness. Depression is more typically thought of as strictly biochemical-based or emotionally-rooted. On the contrary, nutrition can play a key role in the onset as well as severity and duration of depression. Many of the easily noticeable food patterns that precede depression are the same as those that occur during depression. These may include poor appetite, skipping meals, and a dominant desire for sweet foods.[1] Nutritional neuroscience is an emerging discipline shedding light on the fact that nutritional factors are intertwined with human cognition, behavior, and emotions.

The most common mental disorders that are currently prevalent in numerous countries are depression, bipolar disorder, schizophrenia, and obsessive-compulsive disorder (OCD).[2] The dietary intake pattern of the general population in many Asian and American countries reflects that they are often deficient in many nutrients, especially essential vitamins, minerals, and omega-3 fatty acids.[3] A notable feature of the diets of patients suffering from mental disorders is the severity of deficiency in these nutrients.[3] Studies have indicated that daily supplements of vital nutrients are often effective in reducing patients' symptoms.[4] Supplements containing amino acids have also been found to reduce symptoms, as they are converted to neurotransmitters which in turn alleviate depression and other mental health problems.[4] On the basis of accumulating scientific evidence, an effective therapeutic intervention is emerging, namely nutritional supplement/treatment. These may be appropriate for controlling and to some extent, preventing depression, bipolar disorder, schizophrenia, eating disorders and anxiety disorders, attention deficit disorder/attention deficit hyperactivity disorder (ADD/ADHD), autism, and addiction.[4] Most prescription drugs, including the common antidepressants lead to side effects.[4] This usually causes the patients to skip taking their medications. Such noncompliance is a common occurrence encountered by psychiatrists. An important point to remember here is that, such noncompliant patients who have mental disorders are at a higher risk for committing suicide or being institutionalized. In some cases, chronic use or higher doses may lead to drug toxicity, which may become life threatening to the patient.[4] An alternate and effective way for psychiatrists to overcome this noncompliance is to familiarize themselves about alternative or complementary nutritional therapies. Although further research needs to be carried out to determine the best recommended doses of most nutritional supplements in the cases of certain nutrients, psychiatrists can recommend doses of dietary supplements based on previous and current efficacious studies and then adjust the doses based on the results obtained by closely observing the changes in the patient.[4]

When we take a close look at the diet of depressed people, an interesting observation is that their nutrition is far from adequate. They make poor food choices and selecting foods that might actually contribute to depression. Recent evidence suggests a link between low levels of serotonin and suicide.[5] It is implicated that lower levels of this neurotransmitter can, in part, lead to an overall insensitivity to future consequences, triggering risky, impulsive and aggressive behaviors which may culminate in suicide, the ultimate act of inwardly directed impulsive aggression.

Depression is a disorder associated with major symptoms such as increased sadness and anxiety, loss of appetite, depressed mood, and a loss of interest in pleasurable activities. If there is no timely therapeutic intervention, this disorder can lead to varied consequences. Patients who are suffering from depression exhibit suicidal tendency to a larger degree and hence are usually treated with antidepressants and/or psychotherapy.[6] Deficiencies in neurotransmitters such as serotonin, dopamine, noradrenaline, and γ-aminobutyric acid (GABA) are often associated with depression.[6–11] As reported in several studies, the amino acids tryptophan, tyrosine, phenylalanine, and methionine are often helpful in treating many mood disorders including depression.[12–17] When consumed alone on an empty stomach, tryptophan, a precursor of serotonin, is usually converted to serotonin. Hence, tryptophan can induce sleep and tranquility. This implies restoring serotonin levels lead to diminished depression precipitated by serotonin deficiencies.[8] Tyrosine and sometimes its precursor phenylalanine are converted into dopamine and norepinephrine.[18]

Dietary supplements containing phenyl alanine and/or tyrosine cause alertness and arousal. Methionine combines with adenosine triphosphate (ATP) to produce S-adenosylmethionine (SAM), which facilitates the production of neurotransmitters in the brain.[19–22] The need of the present paradigm is, more studies shedding light on the daily supplemental doses of these neurochemicals that should be consumed to achieve antidepressant effects. Researchers attribute the decline in the consumption of omega-3 fatty acids from fish and other sources in most populations to an increasing trend in the incidence of major depression.[23] The two omega-3 fatty acids, eicosapentaenoic acid (EPA) which the body converts into docosahexanoic acid (DHA), found in fish oil, have been found to elicit antidepressant effects in human. Many of the proposed mechanisms of this conversion involve neurotransmitters. For instance, antidepressant effects may be due to bioconversion of EPA to leukotrienes, prostaglandins, and other chemicals required by the brain. Others hypothesize that both EPA and DHA influence neuronal signal transduction by activating peroxisomal proliferator-activated receptors (PPARs), inhibiting G-proteins and protein kinase C, in addition to calcium, sodium, and potassium ion channels. Whichever may be the case, epidemiological data and clinical studies have clearly shown that omega-3 fatty acids can effectively treat depression.[24] In depressed patients, daily consumption of dietary supplements of omega-3 fatty acid that contain 1.5-2 g of EPA has been shown to stimulate mood elevation. Nevertheless, doses of omega-3 higher than 3 g do not show better effects than placebos and may be contraindicative in cases, such as those taking anticlotting drugs.[25] In addition to omega–3 fatty acids, vitamin B (e.g., folate) and magnesium deficiencies have been linked to depression.[26–28]

Randomized, controlled trials that involve folate and vitamin B12 suggest that patients treated with 0.8 mg of folic acid/day or 0.4 mg of vitamin B12/day will exhibit decreased depression symptoms.[27] In addition, the results of several case studies where patients were treated with 125-300 mg of magnesium (as glycinate or taurinate) with each meal and at bedtime led to rapid recovery from major depression in < 7 days for most of the patients. Previous research has revealed the link between nutritional deficiencies and some mental disorders.[23,25,29–32]

The most common nutritional deficiencies seen in patients with mental disorders are of omega–3 fatty acids, B vitamins, minerals, and amino acids that are precursors to neurotransmitters.[20,23,24,27,28,30,33] Accumulating evidence from demographic studies indicates a link between high fish consumption and low incidence of mental disorders; this lower incidence rate being the direct result of omega–3 fatty acid intake.[23,31,32] One to two grams of omega-3 fatty acids taken daily is the generally accepted dose for healthy individuals, but for patients with mental disorders, up to 9.6 g has been shown to be safe and effective.[34–36] Majority of Asian diets are usually also lacking in fruits and vegetables, which further lead to mineral and vitamin deficiencies. The significance of various nutrients in mental health, with special relevance to depression has been discussed below.

## CARBOHYDRATES

Carbohydrates are naturally occurring polysaccharides and play an important role in structure and function of an organism. In higher organisms (human), they have been found to affect mood and behavior. Eating a meal which is rich in carbohydrates triggers the release of insulin in the body. Insulin helps let blood sugar into cells where it can be used for energy and simultaneously it triggers the entry of tryptophan to brain. Tryptophan in the brain affects the neurotransmitters levels.

Consumption of diets low in carbohydrate tends to precipitate depression, since the production of brain chemicals serotonin and tryptophan that promote the feeling of well being, is triggered by carbohydrate rich foods. It is suggested that low glycemic index (GI) foods such as some fruits and vegetables, whole grains, pasta, etc. are more likely to provide a moderate but lasting effect on brain chemistry, mood, and energy level than the high GI foods - primarily sweets - that tend to provide immediate but temporary relief.

## PROTEINS

Proteins are made up of amino acids and are important building blocks of life. As many as 12 amino acids are manufactured in the body itself and remaining 8 (essential amino acids) have to be supplied through diet. A high quality protein diet contains all essential amino acids. Foods rich in high quality protein include meats, milk and other dairy products, and eggs. Plant proteins such as beans, peas, and grains may be low in one or two essential amino acids. Protein intake and in turn the individual amino acids can affect the brain functioning and mental health. Many of the neurotransmitters in the brain are made from amino acids. The neurotransmitter dopamine is made from the amino acid tyrosine and the neurotransmitter serotonin is made from the tryptophan.[5] If there is a lack of any of these two amino acids, there will not be enough synthesis of the respective neurotransmitters, which is associated with low mood and aggression in the patients. The excessive buildup of amino acids may also lead to brain damage and mental retardation. For example, excessive buildup of phenylalanine in the individuals with disease called phenylketonuria can cause brain damage and mental retardation.

## ESSENTIAL FATTY ACIDS

### Omega-3 fatty acids

The brain is one of the organs with the highest level of lipids (fats). Brain lipids, composed of fatty acids, are structural constituents of membranes. It has been estimated that gray matter contains 50% fatty acids that are polyunsaturated in nature (about 33% belong to the omega-3 family), and hence are supplied through diet. In one of the first experimental demonstrations of the effect of dietary substances (nutrients) on the structure and function of the brain, the omega-3 fatty acids (specially alpha-linolenic acid, ALA) were the member to take part. An important trend has been observed from the findings of some recent studies that lowering plasma cholesterol by diet and medications increases depression. Among the significant factors involved are the quantity and ratio of omega-6 and omega-3 polyunsaturated fatty acids (PUFA) that affect serum lipids and alter the biochemical and biophysical properties of cell membranes. It has been hypothesized that sufficient long chain PUFAs, especially DHA, may decrease the development of depression.[37] The structural and functional components of membrane in cells of brain which is a lipid-rich organ, include polar phospholipids, spingolipids, and cholesterol. The glycerophospholipids in brain consist of high proportion of PUFA derived from the essential fatty acids (EFAs), linoleic acid and α-linolenic acid. The main PUFA in the brain are DHA, derived from the omega-3 fatty acid α-linolenic acid, arachidonic acid (AA) and docosa tetraenoic acid, both derived from omega-6 fatty acid linoleic acid. Experimental studies have revealed that diets lacking omega-3 PUFA lead to considerable disturbance in neural function.[38] Studies by Marszalek and Lodish indicate that despite their abundance in the nervous system, DHA and AA cannot be synthesized by mammals *de novo* and hence they or their precursors have to be supplied through the diet and transported to the brain. During late gestation and the early postnatal period, neurodevelopment occurs at significantly rapid rates which make the supply of adequate quantity of PUFAs, particularly DHA, imperative to ensure neurite outgrowth in addition to appropriate development of brain and retina.[39]

Bruinsma and Taren of University of Arizona College of Public Health, Tucson, USA explored the involvement of dieting-related psychological factors as potential confounders.[40] They discussed studies that have both supported and contested the proposition that lowering plasma cholesterol by diet and medications contributes to depression. Research findings point out that an imbalance in the ratio of the EFAs, namely the omega-6 and omega-3 fatty acids, and/or a deficiency in omega-3 fatty acids, may be responsible for the heightened depressive symptoms associated with low plasma cholesterol. These relationships may explain the inconsistency in the results of trials on cholesterol-lowering interventions and depression. On similar lines, dieting behaviors have been associated with alterations in moods.[41] Dietary omega-3 fatty acids play a role in the prevention of some disorders including depression. Their deficiency can accelerate cerebral aging by preventing the renewal of membranes. However, the respective roles of the vascular component on one hand (where the omega-3s are active) and the cerebral parenchyma itself on the other, have not yet been clearly resolved. The role of omega–3 in certain diseases such as dyslexia and autism is suggested. It was omega–3 fatty acids that participated in the first coherent experimental demonstration of the effect of dietary substances (nutrients) on the structure and function of the brain. Experiments were first of all carried out on *x-vivo* cultured brain cells (1), then on *in vivo* brain cells (2), finally on physicochemical, biochemical, physiological, neurosensory, and behavioral parameters (3). These findings indicated that the nature of polyunsaturated fatty acids (in particular omega–3) present in formula milks for infants (both premature and term) determines the visual, cerebral, and intellectual abilities.[16]

## VITAMINS

### B-complex vitamins

Nutrition and depression are intricately and undeniably linked, as suggested by the mounting evidence by researchers in neuropsychiatry. According to a study reported in Neuropsychobiology,[42] supplementation of nine vitamins, 10 times in excess of normal recommended dietary allowance (RDA) for 1 year improved mood in both men and women. The interesting part was that these changes in mood after a year occurred even though the blood status of nine vitamins reached a plateau after 3 months. This mood improvement was particularly associated with improved vitamin B2 and B6 status. In women, baseline vitamin B1 status was linked with poor mood and an improvement in the same after 3 months was associated with improved mood.

Thiamine is known to modulate cognitive performance particularly in the geriatric population.[43]

### Vitamin B12 (Cynocobalamin)

Clinical trials have indicated that Vitamin B12 delays the onset of signs of dementia (and blood abnormalities), if it is administered in a precise clinical timing window, before the onset of the first symptoms. Supplementation with cobalamin enhances cerebral and cognitive functions in the elderly; it frequently promotes the functioning of factors related to the frontal lobe, in addition to the language function of people with cognitive disorders. Adolescents who have a borderline level of vitamin B12 deficiency develop signs of cognitive changes.[43]

### Folate

It has been observed that patients with depression have blood folate levels, which are, on an average, 25% lower than healthy controls.[44] Low levels of folate have also been identified as a strong predisposing factor of poor outcome with antidepressant therapy. A controlled study has been reported to have shown that 500 mcg of folic acid enhanced the effectiveness of antidepressant medication.[45] Folate's critical role in brain metabolic pathways has been well recognized by various researchers who have noted that depressive symptoms are the most common neuropsychiatric manifestation of folate deficiency.[46] It is not clear yet whether poor nutrition, as a symptom of depression, causes folate deficiency or primary folate deficiency produces depression and its symptoms.

## MINERALS

### Calcium

A recent study showed that selective serotonin uptake inhibitors (SSRIs) inhibit absorption of calcium into bones. In addition to this, the SSRIs can also lower blood pressure in people, resulting in falls which may lead to broken bones. Indiscriminate prescription of SSRIs by doctors and ingestion by patients at risk of depression or other mental health problems may put them at increased risk of fractures. Compounded by the fact that they may be aging and already taking other medications, may also predispose them to osteoporosis.[47]

### Chromium

Many studies on the association of chromium in humans depression have been recorded[48,49] which indicate the significance of this micronutrient in mental health.

### Iodine

Iodine plays an important role in mental health. The iodine provided by the thyroid hormone ensures the energy metabolism of the cerebral cells. During pregnancy, the dietary reduction of iodine induces severe cerebral dysfunction, eventually leading to cretinism.

### Iron

Iron is necessary for oxygenation and to produce energy in the cerebral parenchyma (through cytochrome oxidase), and for the synthesis of neurotransmitters and myelin. Iron deficiency is found in children with attention-deficit/hyperactivity disorder. Iron concentrations in the umbilical artery are critical during the development of the foetus, and in relation with the IQ in the child; Infantile anemia with its associated iron deficiency is associated with disturbance in the development of cognitive functions.[43] Research findings pointed out that twice as many women as men are clinically depressed. This gender difference starts in adolescence and becomes more pronounced among married women aged 25-45, with children. Furthermore, women of childbearing age experience more depression than during other times in their lives. These indicate the possible importance of iron in the etiology of depression since its deficiency is known to cause fatigue and depression. Iron deficiency anemia is associated, for instance, with apathy, depression, and rapid fatigue when exercising.[43]

### Lithium

Lithium, a monovalent cation, was first discovered and defined by Johan August in 1817 while he did an analysis of the mineral petalite. The role of lithium has been well known in psychiatry. Half a century into its use, its choice for bipolar disorder with antimanic, antidepressant, and antisuicidal property. The therapeutic use of lithium also includes its usage as an augmenting agent in depression, scizoaffective disorder, aggression, impulse control disorder, eating disorders, ADDs, and in certain subsets of alcoholism.[50] But adequate care has to be taken while using lithium, the gold standard mood stabilizer, in the mentally ill. Lithium can be used in patients with cardiovascular, renal, endocrine, pulmonary, and dermatological comorbidity. The use of lithium during pregnancy and lactation, in pediatric and geriatric population needs careful observation about its toxicity.

### Selenium

In a large review, Dr. David Benton of the university of Wales identified at least five studies, which indicate that low selenium intake is associated with lowered mood status.[51] Intervention studies with selenium with other patient populations reveal that selenium improves mood and diminishes anxiety.[52,53]

### Zinc

Zinc participates among others in the process of gustation (taste perception). At least five studies have shown that zinc levels are lower in those with clinical depression.[54] Furthermore, intervention research shows that oral zinc can influence the effectiveness of antidepressant therapy.[55] Zinc also protects the brain cells against the potential damage caused by free radicals.

Several studies have revealed the full genetic potential of the child for physical development and mental development may be compromised due to deficiency (even subclinical) of micronutrients. When children and adolescents with poor nutritional status are exposed to alterations of mental and behavioral functions, they can be corrected by dietary measures, but only to certain extent. It has been observed that, nutrient composition of diet and meal pattern can have beneficial or adverse, immediate or long-term effects. Dietary deficiencies of antioxidants and nutrients (trace elements, vitamins, and nonessential micronutrients such as polyphenols) during aging may precipitate brain diseases, which may be due to failure for protective mechanism against free radicals.

## OTHER PHYSIOLOGICAL AND PSYCHOSOCIAL FACTORS

Another angle of viewing diet and depression involves old age, which is a time of vulnerability to unintentional weight loss, a factor that is often linked to increased morbidity and premature death. Anorexia of aging may play an important role in precipitating this, by either reducing food intake directly or reducing food intake in response to such adverse factors as age-associated reductions in sensory perception (taste and smell), poor dentition, use of multiple prescription drugs, and depression.[56] Marcus and Berry[57] reviewed malnutrition occurring in the elderly, in both institutional and community settings, due to refusal to eat. They suggest physiologic changes associated with aging, mental disorders such as dementia and depression, and medical, social, and environmental as causative factors. Currently to tackle the problem of depression, people are following the alternative and complementary medicine (CAM) interventions. CAM therapies are defined by the National Center for Complementary and Alternative Medicine as a group of diverse medical and health systems, practices, and products that are not currently considered to be a part of conventional medicine.[58] Mental health professionals need to be aware that it is likely that a fair number of their patients with bipolar disorder might use CAM interventions. Some clinicians judge these interventions to be attractive and safe alternatives, or adjuncts to conventional psychotropic medications.[59]

Current research in psychoneuroimmunology and brain biochemistry indicates the possibility of communication pathways that can provide a clearer understanding of the association between nutritional intake, central nervous system, and immune function thereby influencing an individual's psychological health status. These findings may lead to greater acceptance of the therapeutic value of dietary intervention among health practitioners and health care providers addressing depression and other psychological disorders.
